# Identification of Two Cytochrome Monooxygenase P450 Genes, *CYP321A7* and *CYP321A9*, from the Tobacco Cutworm Moth (*Spodoptera Litura*) and Their Expression in Response to Plant Allelochemicals

**DOI:** 10.3390/ijms18112278

**Published:** 2017-10-30

**Authors:** Rui-Long Wang, Ya-Nan He, Christian Staehelin, Shi-Wei Liu, Yi-Juan Su, Jia-En Zhang

**Affiliations:** 1 Guangdong Engineering Research Center for Modern Eco-Agruculture and Circular Agriculture, Guangzhou 510642, China; rlw2009@scau.edu.cn (R.-L.W.); yananhe94@163.com (Y.-N.H.); 13711379414@163.com (S.-W.L.); syj@scau.edu.cn (Y.-J.S.); 2 Key Laboratory of Agro-Environment in the Tropics, Ministry of Agriculture, South China Agricultural University, Guangzhou 510642, China; 3 State Key Laboratory of Biocontrol and Guangdong Key Laboratory of Plant Resources, School of Life Sciences, Sun Yat-sen University, East Campus, Guangzhou 510006, China; cst@mail.sysu.edu.cn; 4 Key Laboratory of Agroecology and Rural Environment of Guangdong Regular Higher Education Institutions, South China Agricultural University, Guangzhou 510642, China

**Keywords:** cytochrome P450 monooxygenases, *Spodoptera litura*, plant allelochemicals, *CYP321A7*, *CYP321A9*

## Abstract

Larvae of the polyphagous tobacco cutworm moth, *Spodoptera litura* (*S. litura*)*,* encounter potentially toxic allelochemicals in food. It is therefore important for *S. litura* to produce detoxification enzymes such as cytochrome P450 monooxygenases (P450s). In this study, we have identified two novel cytochrome P450 genes of *S. litura*, named *CYP321A7* and *CYP321A9.* Phylogenetic analysis indicated that they belong to the *CYP321A* subfamily. Expression levels of these genes at different development stages were determined by real-time quantitative polymerase chain reaction (PCR). The highest expression was found in the midgut and the fat body. Larvae fed with a diet supplemented with xanthotoxin or coumarin showed a strongly increased expression of *CYP321A7* and *CYP321A9* in the midgut and fat body as compared to larvae that consumed a control diet. In contrast, larvae consuming a diet containing aflatoxin B1 or quercetin did not induce the expression of these genes. *CYP321A7* and *CYP321A9* showed different expression profiles with respect to certain allelochemicals. For example, a diet containing cinnamic acid stimulated the expression of *CYP321A9*, whereas no changes were observed for *CYP321A7*. We suggest that the fine tuning of P450 gene expression is an important adaptation mechanism that allows polyphagous *S. litura* larvae to survive in a changing chemical environment.

## 1. Introduction

The tobacco cutworm moth, *Spodoptera litura* (Fabricius) is a noctuid moth (Lepidoptera: Noctuidae), which is also known as the common cutworm, cluster caterpillar, cotton leafworm or tropical armyworm. *S. litura* is a polyphagous pest of many crop plants and is widely distributed throughout the world [1]. *S. litura* causes damage to more than 99 different families of plants and approximately 209 species, including important crop plants such as tobacco (*Nicotiana tabacum*), cabbage (*Brassica oleracea*), soybean (*Glycine max*), tomato (*Lycopersicon esculentum*) and cotton (*Gossypium hirsutum*) [1,2]. Herbivorous insects are frequently exposed to toxic secondary metabolites produced by host plants. Insects therefore adjust the expression level of detoxification genes to cope with these allelochemicals. The perception of specific allelochemicals and activation of signal transduction pathways, culminating in the expression of specific detoxification genes, is a well-known adaptation strategy to a given host plant [3]. Up-regulation of detoxification genes helps polyphagous insects to consume different types of food from different host plants [3,4,5,6,7]. The polyphagous *S. litura* larvae must cope with a particularly broad range of toxic plant allelochemicals in the consumed food [6]. It is therefore important for *S. litura* to evolve adaptation mechanisms to the chemical composition of a given host plant.

Cytochrome P450 monooxygenases (P450s or CYPs) constitute a large superfamily of heme-thiolate enzymes that are involved in the oxidative metabolism of endogenous and exogenous compounds [3,8]. Insect P450s have been divided into four clades: CYP2, CYP3, CYP4 and the mitochondrial CYP clade [3]. The clade *CYP3* is further subdivided into different *CYP* families. The *CYP321* subfamily is well known to play a role in the metabolism of xenobiotics and insecticide resistance [3,4]. In the corn earworm *(Helicoverpa zea)* for example, the P450s CYP321A1 and CYP6B8 have been found to metabolize plant allelochemicals, such as xanthotoxin and angelicin [4,9]. Accordingly, the expression of the *CYP321A1* gene in the midgut of *H. zea* larvae was considerably increased in response to xanthotoxin, coumarin and indol 3-carbinol [10]. Furthermore, expression levels of the *CYP6B8* and *CYP6B28* genes, which belong to the CYP3 clade, were significantly increased when *H. zea* larvae were fed with a diet containing xanthotoxin, phenobarbital, indole 3-carbinol, chlorogenic acid, rutin or flavones, suggesting a role for these P450 genes in the detoxification of plant-derived allelochemicals [11]. In other studies on *H. zea*, *CYP6B8* expression levels were elevated in response to a diet containing xanthotoxin, coumarin, flavone, visnagin and imperatorin [10,12]. In larvae of the cotton bollworm (*Helicoverpa armigera*), expression levels of the *CYP6B6* gene correlated with the concentration of 2-tridecanone. Expression levels of *CYP6B6* in the midgut and fat body were also elevated when larvae were fed with a mixture of 2-tridecanone and quercetin in their diet [13]. Likewise, the expression of *CYP321A1* in *H. armigera* and *CYP6B1* in *Papilio polyxenes* was induced by xanthotoxin in order to detoxify this allelochemical [14]. The activity of P450s in the midgut of the silkworm (*Bombyx mori*) was stimulated 2.3-fold after feeding larvae with diet containing quercetin [15]. The expression of *CYP6A8, CYP6D5, CYP6W1, CYP9B2,* and *CYP12D1* genes in *Drosophila melanogaster* was induced by black pepper *(Piper nigrum)* extracts [16]*.* In *Aedes aegypti*, the expression of *CYP4*, *CYP6* and *CYP9* family genes was significantly induced in response to different combinations of allelochemicals in leaf litter [17]. The expression of *CYP6A2* and *CYP6A8 D. melanogaster* was found to be induced by caffeine [18]. In contrast, the expression of *CYP4M18*, *CYP4M14* and *CYP9A28* in *Spodoptera frugiperda* was low when the larvae consumed xanthotoxin [4]. Likewise, the expression of *CYP6A8* in the fat body of *S. litura* was significantly reduced when larvae were fed with coumarin and cinnamic acid [6].

Similar to these reports, expression levels of *Spodoptera* P450 genes were influenced by consumed plant allelochemicals and insecticides. In *S. litura, CYP6AB14* expression was induced when larvae were eating a diet containing xanthotoxin, coumarin or flavones [2]. In the midgut, expression levels of *CYP6B58* were significantly enhanced by coumarin (1.5-fold) and xanthotoxin (1.7-fold) in the diet. The expression of *CYP6B48* in the midgut was increased in response to consumed flavone (21.6-fold) or xanthotoxin (18.2-fold) [6]. *CYP9A40* expression in the midgut significantly increased in response to the uptake of quercetin (6.5-fold) or cinnamic acid (5.3-fold) [19]. Expression levels of *CYP321B1* were recently found to play a role in the detoxification of various insecticides [20]. Moreover, the expression of *CYP321A* family genes was found to be induced by allelochemicals in another *Spodoptera* species (fall armyworm; *S. frugiperda*). Expression levels of *CYP321A7*, *CYP321A8* and *CYP321A9* were strongly induced in the midgut in response to xanthotoxin and other consumed allelochemicals [14]. In summary, these findings indicate that the expression of P450 genes in *Spodoptera* larvae is regulated by the consumption of xenobiotics, and suggest that these P450s are involved in the detoxification of allelochemicals and/or insecticides, which helps the *Spodoptera* larvae to adapt to the chemical environment around them.

Understanding insect responses to plant allelochemicals and pesticides in their local ecological context provides key information for the development of pest control strategies [21]. This includes knowledge of P450 genes in *S. litura*, and the impact of plant allelochemicals on their expression profiles. In this study, two novel P450 genes of *S. litura,* named *CYP321A7* and *CYP321A9,* were identified using a whole transcriptome sequencing approach. Quantitative real-time PCR (RT-qPCR) was used to investigate the tissue- and developmental-specific expression of these P450 genes. We further examined whether these genes are potentially involved in the metabolism of plant allelochemicals. Larvae were fed with a diet supplemented with ten different plant allelochemicals. RT-qPCR analysis showed that the expression of *CYP321A7* and *CYP321A9* in the midgut and fat body was up-regulated in response to specific allelochemicals, suggesting a role of these P450s in the oxidative detoxification of plant allelochemicals.

## 2. Results

### 2.1. Identification of Two Novel Cytochrome P450 Genes Expressed in S. litura

The whole transcriptome shotgun sequencing data for *S. litura* midgut RNA from fourth instar larvae were analyzed on the Illumina sequencing platform. In total, 33,447,158 reads assembled into 76,160 contigs, and 28,936 unigenes were obtained. Using the BLASTX algorithm, all unigenes were compared with NCBI-nr, Swiss-Prot, GO, COGs, KOG and KEGG protein databases. The transcriptome analysis indicated the expression of 23 different P450 genes, along with six esterase and three glutathione S-transferase genes (see Table S1). Using *S. litura* midgut cDNA, the full-length coding sequences of two novel P450 genes of *S. litura*—*CYP321A7* (GenBank Accession No. MF802804) and *CYP321A9* (GenBank Accession No. MF802805)—were PCR-cloned and confirmed by sequencing.

**Figure 1:**
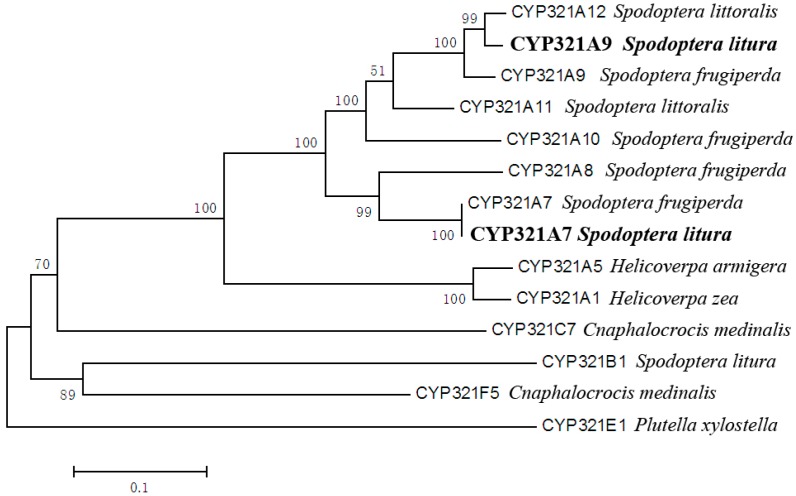
Phylogenetic analysis of CYP321A7 and CYP321A9 of *Spodoptera litura* and related P450s. The phylogenetic tree was constructed with MEGA 6.0 software (Neighbour-joining method). The scale bar indicates 0.1 amino acid substitutions per site. Bootstrap analysis was performed with 1000 replications.

Primary sequence alignments with related P450 genes revealed that *CYP321A7* and *CYP321A9* belong to the *CYP321A* subfamily of the *CYP321* gene family. Within the *CYP321A* subfamily, the predicted *S. litura* CYP321A7 and CYP321A9 amino acid sequences are most related to those encoded by genes from other *Spodoptera* species. CYP321A7 is most similar to *S. frugiperda* CYP321A7 (96.9% amino acid identity), *S. frugiperda* CYP321A8 (85.3% identity) and *S. frugiperda* CYP321A9 (81.2% identity). The predicted CYP321A9 protein sequence shares high amino acid identity with *S. littoralis* CYP321A12 (96.6% identity), *S. frugiperda* CYP321A9 (95.2% identity) and *S. littoralis* CYP321A11 (88.5% identity). A constructed phylogenetic tree is shown in Figure 1.

The *CYP321A7* cDNA contained an open reading frame (ORF) of 1488 base pair (bp), which corresponds to a predicted protein of 495 amino acid residues, with a calculated molecular weight of 56.89 kDa and a theoretical isoelectric point of 9.14. The SignalP 4.1 program predicted that the first 17 N-terminal amino acid residues of CYP321A7 represent a signal peptide, suggesting a membrane-associated protein. The cDNA sequence of *CYP321A9* contained an ORF of 1494 bp encoding a predicted polypeptide of 497 amino acid residues, with a calculated molecular weight of 57.13 kDa and a theoretical isoelectric point of 8.66. An N-terminal signal peptide sequence of 17 amino acids was also found for CYP321A9, suggesting that this P450s is also a membrane-bound protein.

**Figure 2:**
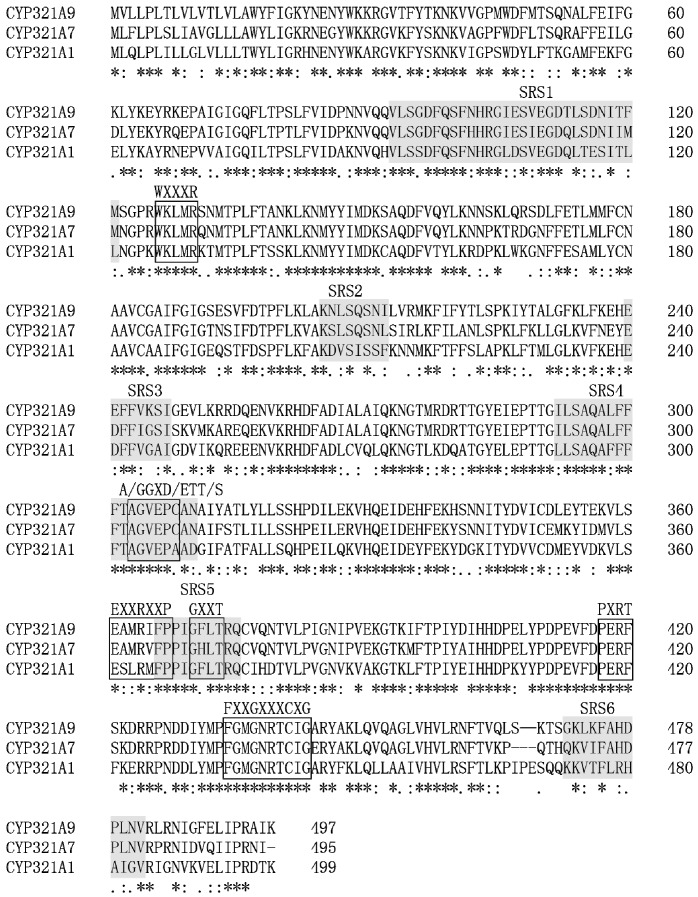
Alignment of the amino acid sequences deduced from CYP321A7 (*Spodoptera litura*), CYP321A9 (*S. litura*) and CYP321A1 (*Helicoverpa zea*). Conserved motifs (WXXXR, GXXT, EXXRXXP, PXRF, FXXGXXXCXG and A/GGXD/ETT/S) of cytochrome P450 proteins are boxed. Substrate recognition sites (SRS) are marked in gray. Fully conserved residues are marked by asterisks, highly similar residues by double dots and moderately similar residues by single dots. Accession numbers: MF802804 for CYP321A7, MF802805 for CYP321A9 and AY113689.1 for CYP321A1. Conserved amino acid residues are indicated below: “*” means a single, fully conserved residue; “:” indicates a strongly and “.” a weakly conserved residue.

A protein sequence alignment for *S. litura* CYP321A7, *S. litura* CYP321A9 and the closely related *H. zea* CYP321A1 protein is shown in Figure 2. CYP321A7 contains several conserved motifs that are characteristic of P450 genes [3,20,22], namely the C-helix sequence motif WXXXR (WRLIR at positions 126–130), the K-helix motif EXXRXXP (EAMRVFP at positions 361–367), the threonine-containing binding pocket motif A/GGXD/ETT/S (AGVEPC at positions 303–308), the heme-binding motif FXXGXXXCXG (FGMGNRTCIG at positions 444–453), and the putative “meander”-binding sequences EXXR and PXXF (EAMR at positions 361–364 and PERF at positions 417–420) (Figure 2). The conserved P450 motifs in the CYP321A9 protein sequence are identical to those indentified in the CYP321A7 sequence. The protein sequence alignment also indicates the presence of six predicted substrate recognition sites (SRSs) (SRSs; SRS1 to SRS6 in Figure 2) that are conserved in the *CYP321* family of lepidopteran insects [9].

### 2.2. Tissue-Dependent Expression Pattern of *CYP321A7* and *CYP321A9*

**Figure 3:**
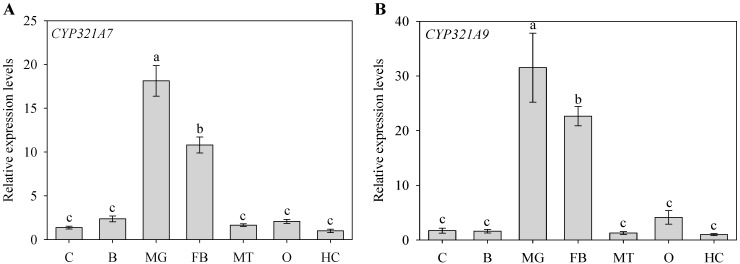
Expression of *CYP321A7* (**A**) and *CYP321A9* (**B**) in different tissues of 2-day-old fourth instar larvae of *Spodoptera litura*. Analyzed tissues: cuticle (C), brain (B), midgut (MG), fat body (FB), Malpighian tubule (MT), ovary (O) and hemocytes (HC). Reverse transcription–qPCR (RT–qPCR) analysis was used to determine the expression of *CYP321A7* and *CYP321A9* as described in the Experimental Section. The *β*-actin gene served as an internal reference to determine relative expression levels. Data shown are means ± standard error (SE) (three biological replicates). Different letters above bars indicate significant differences (*p* < 0.05) according to the Duncan’s multiple range test.

Expression levels of *CYP321A7* and *CYP321A9* in seven different tissues (cuticle, brain, midgut, fat body, Malpighian tubule, ovary and hemolymph) were determined by RT-qPCR. The obtained data showed that both genes were expressed in all examined tissues, but expression levels varied depending on the tissue (Figure 3). The expression of *CYP321A7* was particularly high in the midgut and in the fat body. Expression levels in the midgut were 1.7- to 18.1-fold higher than those in the other tissues (Figure 3A). A similar expression pattern with highest values for the midgut and fat body was obtained for *CYP321A9.* Only low expression levels were observed in the cuticle, brain, ovary and hemolymph. The expression levels of *CYP321A9* in the midgut were 1.4- to 31.5-fold higher than those in the other tissues (Figure 3B).

### 2.3. Stage-Dependent Expression Pattern of *CYP321A7* and *CYP321A9*

**Figure 4:**
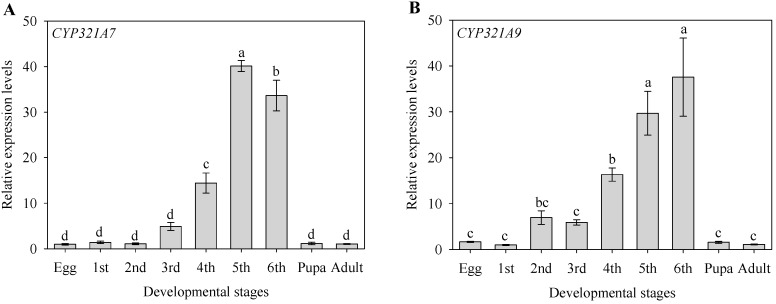
Expression of *CYP321A7* (**A**) and *CYP321A9* (**B**) at different developmental stages of *Spodoptera litura*. Instars are abbreviated (1st to 6th). Data were obtained by RT-qPCR as described in the Experimental Section. The β-actin gene was used as an internal reference to determine relative expression levels. Data shown are means ± SE (three biological replicates). Different letters above bars indicate significant differences (*p* < 0.05) according to the Duncan’s multiple range test.

The expression levels of *CYP321A7* and *CYP321A9* also significantly varied among the nine life stages of *S. litura* (i.e., eggs, the six instars, pupae and the adult stage) (Figure 4)*.* The highest expression levels of *CYP321A7* were detected in the fifth and sixth instar larvae of *S. litura*. Values for the fifth instar were 1.2- to 40.1-fold higher than for other stages, whereas low levels were observed in eggs, first and second instars, pupae and adults (Figure 4A). Expression data for *CYP321A9* were similar to those of *CYP321A7.* The highest expression levels were found in fifth and sixth instars with values 1.3- to 37.6-fold higher than in the other stages (Figure 4B).

### 2.4. Altered Expression of *CYP321A7* and *CYP321A9* in Response to Plant Allelochemcials

**Figure 5:**
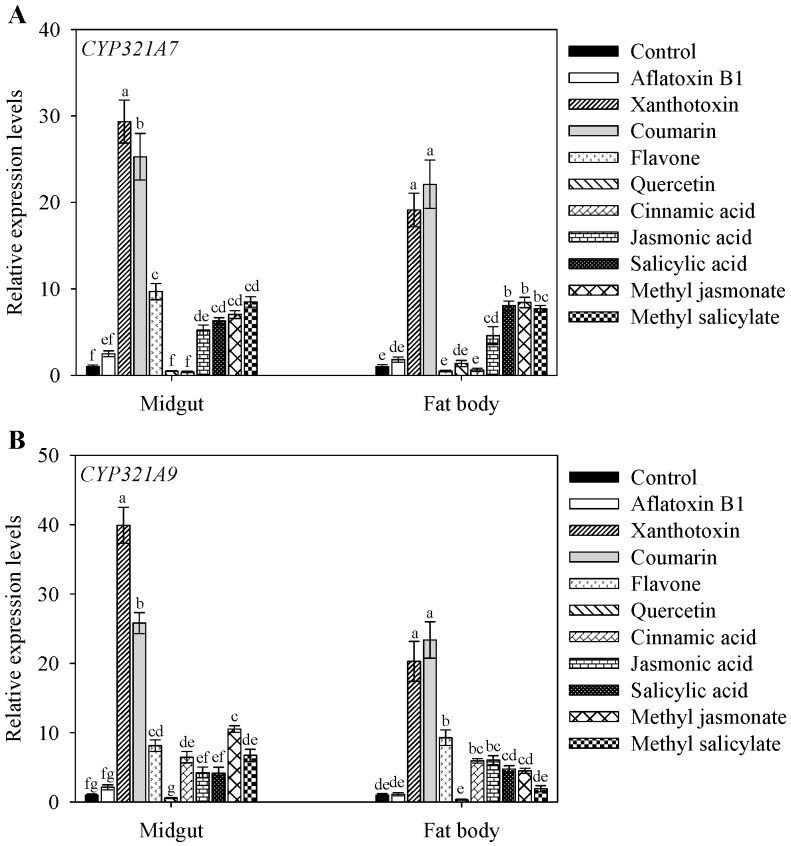
Expression levels of *CYP321A7* (**A**) and *CYP321A9* (**B**) in *Spodoptera litura* after feeding fourth instar larvae with a diet containing different allelochemicals. The larvae were reared on a diet without allelochemicals (control) or on different diets containing 1.0 μg·g^−1^ aflatoxin B1, 0.25 mg·g^−1^ xanthotoxin, 1.0 mg·g^−1^ coumarin, 1.0 mg·g^−1^ flavone, 1.0 mg·g^−1^ quercetin, 1.0 mg·g^−1^ cinnamic acid, 2.9 μg·g^−1^ jasmonic acid, 12.0 μg·g^−1^ salicylic acid, 2.9 μg·g^−1^ methyl jasmonate or 12.0 μg·g^−1^ methyl salicylate. RNA from midguts and fat bodies was extracted after the larvae had consumed the diet for 48 h. RT-qPCR analysis was used to determine the relative expression levels of *CYP321A7* and *CYP321A9*. The *β*-actin gene served as an internal reference. Data shown are means ± SE (three biological replicates). Different letters above bars indicate significant differences (*p* < 0.05) according to the Duncan’s multiple range test.

To determine the effect of plant allelochemicals on *CYP321A7* and *CYP321A9* expression in *S. litura*, RT-qPCR was performed after feeding 2-day-old fourth instar larvae for 48 h with an artificial diet supplemented with aflatoxin B1, xanthotoxin, coumarin, flavone, quercetin, cinnamic acid, jasmonic acid, salicylic acid, methyl jasmonate or methyl salicylate. Compared to larvae fed with a control diet without allelochemicals, the expression of *CYP321A7* in the midgut was significantly enhanced after consumption of xanthotoxin (29.3-fold), coumarin (25.3-fold), flavones (9.7-fold), methyl salicylate (8.5-fold), methyl jasmonate (7.0-fold), salicylic acid (6.3-fold) and jasmonic acid (5.2-fold). In contrast, the diet containing aflatoxin B1, quercetin or cinnamic acid showed no significant effects on *CYP321A7* expression in the midgut. The expression of *CYP321A7* in the fat body was increased by a diet supplemented with xanthotoxin (22.1-fold), coumarin (19.1-fold), jasmonic acid (4.6-fold), salicylic acid (8.1-fold), methyl jasmonate (8.4-fold) and methyl salicylate (7.7-fold), whereas no significant changes were measured with diet supplementation of aflatoxin B1, flavone, quercetin and cinnamic acid (Figure 5A).

The gene expression pattern of *CYP321A9* was different from *CYP321A7.* Compared to the control (diet without allelochemicals), *CYP321A9* expression in the midgut was increased in response to a diet supplemented with xanthotoxin (39.9-fold), coumarin (25.8-fold), flavone (8.1-fold), cinnamic acid (6.5-fold), methyl jasmonate (10.5-fold) and methyl salicylate (6.7-fold). Aflatoxin B1, quercetin, jasmonic acid and salicylic acid did not significantly affect *CYP321A9* expression in the midgut. The expression of *CYP321A9* in the fat body showed a similar pattern (Figure 5B). A significantly increased induction of *CYP321A9* expression was measured for diets containing coumarin (23.4-fold), xanthotoxin (20.3-fold) and flavone (9.3-fold).

## 3. Discussion

To cope with high amounts of allelochemicals, insects can activate specific P450 genes to metabolize plant allelochemicals more efficiently [3,20,22,23,24]. Induction of Lepidoptera (e.g., in *H. zea*, *S. litura* and *S. frugiperda*) P450 gene expression by plant allelochemicals such as flavone, xanthotoxin, coumarin, indole and indole 3-carbinol has been reported in various studies [6,10,12,14,23]. Here, we have identified *CYP321A7* and *CYP321A9,* two novel P450 genes of *S. litura* belonging to the *CYP321* gene family (*CYP321A* subfamily). Our work provides information on the constitutive expression of *CYP321A7* and *CYP321A9* in different tissues and developmental stages. In addition, we provide experimental evidence that the expression of these genes in larval midguts and fat bodies is considerably up-regulated in response to specific plant allelochemicals, particularly xanthotoxin and coumarin.

Conserved SRSs in P450 protein sequences can provide clues to possible substrate metabolism [25]. The SRSs in *H. zea* CYP321A1, a protein known to be involved in the metabolism of plant allelochemicals and insecticides [9,22], show homology with the predicted SRSs of CYP321A7 and CYP321A9 of *S. litura* (Figure 2). The amino acid residues of SRS1, SRS4, and SRS5 are apparently most conserved in CYP321A7 and CYP321A9. Their similarities (amino acid identity) to *H. zea* CYP321A1 are ranging from 62.1% to 90.0% for CYP321A7 and 69.0% to 100.0% for CYP321A9. Based on these similarities, we suggested that CYP321A7 and CYP321A9 show enzyme properties related to *H. zea* CYP321A1 (i.e., that these three genes are likely involved in the detoxification of similar plant allelochemicals). Moreover, these SRS similarities predict that CYP321A7 and CYP321A9 could also play a role in P450-mediated oxidation of insecticides as shown for *H. zea* CYP321A1 [9,10,22] and other *S. litura* P450s such as *CYP9A40* [19], *CYP6AB14* [2] and *CYP321B1* [20].

To provide clues on the function of *CYP321A7* and *CYP321A9*, we used RT-qPCR to analyze the expression patterns of *CYP321A7* and *CYP321A9* in various tissues of fourth instar larvae and in different developmental stages of *S. litura*. Expression levels of *CYP321A7* and *CYP321A9* were highest in the larval midgut and fat body (Figure 3). Various studies have shown that midguts and fat bodies of insect larvae are main tissues for metabolism, where ingested plant allelochemicals can be efficiently detoxified prior to food adsorption [3,6]. However, other insect P450s can be predominantly expressed in other tissues. For example, *CYP6BQ9* of *Tribolium castaneum,* which is involved in resistance to the insecticide deltamethrin, is strongly expressed in the brain. Identification of such brain-specific insect P450s provides new light on molecular mechanisms underlying insecticide resistance [26]. *CYP321A7* and *CYP321A9*, analyzed in this study, may be involved in the metabolism of endogenous or exogenous materials in the midgut and fat body. Our expression data are reminiscent of those from other P450 genes in *S. litura*, namely the constitutive expression of *CYP6B48*, *CYP6B58*, *CYP6AB14*, *CYP9A40* and *CYP321B1* in the midgut and fat body [2,6,19,20]. Strong expression of P450 genes in the absence of allelochemicals has been also reported for related species, namely *CYP321A7*, *CYP321A8*, *CYP321A9* and *CYP321A10* in the midgut and fat body of *S*. *frugiperda* [14] and *CYP321A1* in the midgut of *H. zea* [9,10].

Polyphagous insects are well adapted to the constantly changing plant toxins in their diets, and P450s play an important role in detoxification processes [14,19,27,28,29,30]. P450s are key mediators of the hydroxylation and epoxidation reactions required for the efficient destruction and elimination of toxins prior to their adsorption in gut tissues [28,30]. Our feeding experiment showed that expression levels of *CYP321A7* and *CYP321A9* are up-regulated when larvae were consuming diet that was supplemented with specific allelochemicals such as xanthotoxin and coumarin (Figure 5). These findings suggest that *CYP321A7* and *CYP321A9* are probably involved in the detoxification of these plant compounds and that the observed induction of gene expression represents an adaptation strategy to cope with high toxin levels. A similar induction of P450s by xanthotoxin and/or coumarin in the diet has been previously reported in various studies on other P450s genes. Examples of genes with increased expression in response to xanthotoxin and/or coumarin are *CYP6AB14* in *S. litura* [2], *CYP321A1* in *H. zea* [9], and *CYP321A7*, *CYP321A8* and *CYP321A9* in *S. frugiperda* [14].

Remarkably, expression levels of *CYP321A7* and *CYP321A9* in the larvae feeding experiment were not affected by certain allelochemicals. Quercetin, for example, had no induction effect, or perhaps even a negative effect, on the gene expression of *CYP321A7* and *CYP321A9.* This is reminiscent of previous studies in which negative effects of allelochemicals on P450 gene expression were reported. For example, *CYP6B1* expression of *P. polyxenes* could be inhibited by various compounds, including flavonoids, alkaloids, coumarins and furanochromones [31]. Likewise, gossypol (at 2 mg·g^−1^) significantly suppressed the expression of *CYP9A12* (2.1-fold) and *CYP9A17* (1.9-fold) in the midgut of *H. armigera* [32].

In contrast to *CYP321A7*, the expression of *CYP321A9* in *S. litura* was differentially affected by consumed plant allelochemicals. Most remarkably, expression levels of *CYP321A9* in the midgut and fat body were significantly induced by a diet containing cinnamic acid, whereas no changes were observed for *CYP321A7* (Figure 5). These findings suggest that *CYP321A9* plays a role in the breakdown of cinnamic acid. Only a single concentration of cinnamic acid was tested in our study, however. Higher doses of cinnamic acid are perhaps necessary to induce *CYP321A7* expression. It is worth mentioning that the doses of allelochemicals used in our study were defined based on available references and according to reported toxicity test results [14].

In conclusion, this study reports on the identification and expression of the *S. litura* genes *CYP321A7* and *CYP321A9*. Induction of these genes by specific allelochemicals differed in these closely related genes, suggesting that fine tuning of P450 gene expression is an important mechanism that allows larvae to survive in a changing chemical environment. P450 genes of *S. litura* are expected to play a role in the choice of host plants. Knowledge of the adaptation of *S. litura* to specific plant allelochemicals and their P450-mediated detoxification mechanisms forms the scientific basis for effective pest control, as many P450 genes are also involved in the destruction of pesticides [3,5,19,20]. Future experiments will be required in order to analyze the expression of *CYP321A7* and *CYP321A9* in response to insecticides, and to address the question of whether these genes play a role in insecticide resistance.

## 4. Experimental Section

### 4.1. Insect Rearing

The *S. litura* used in this study were derived from individuals obtained in July 2015 from a test field on the campus of the South China Agricultural University (SCAU) in Guangzhou, China (23°16′ N, 113°34′ E). Larvae were obtained from a colony maintained by the SCAU Department of Ecology. The larvae were reared in an insectary (25 ± 2 °C and 70 ± 5% relative humidity at a light: dark photoperiod of 14:10 h) and fed with an artificial diet [33]. *S. litura* has been maintained for over 15 generations.

### 4.2. Identification of *CYP321A7* and *CYP321A9* by Whole Trancriptome Analysis

Whole transcriptome shotgun sequencing was performed for *S. litura* midgut RNA from fourth instar larvae. Total *S. litura* RNA was isolated with Trizol according to the manufacture’s protocol (Invitrogen/ThermoFisher Scientific, Waltham, MA, USA) and monitored on 1% agarose gels. RNA quantity was assessed using NanoDrop ND2000 (NanoDrop Technologies, Wilmington, DE, USA) and Qubit 2.0 (Thermo Fisher Scientific, MA, USA). RNA integrity was evaluated with Agilent 2100 Bioanalyzer (Agilent Technologies, Waldbronn, Germany). Three μg of total RNA was used to isolate poly (A) mRNA and to prepare a nondirectional Illumina RNA-Seq library with the Illumina TruSeq™ RNA Sample Preparation Kit (Illumina, San Diego, CA, USA) following the manufacturer’s recommendations. The library was sequenced on an Illumina MiSeq platform at Guangdong Magigene Biotechnology Co., Ltd., Guangzhou, China. The results obtained provided sequence information on *CYP321A* subfamily genes, named *CYP321A7* and *CYP321A9*.

### 4.3. Cloning of *CYP321A7* and *CYP321A9*

To obtain the full-length sequences of the *CYP321A7* and *CYP321A9* genes, mRNA was isolated from the midgut of fouth instar larvae of *S. litura* and cDNA was synthesized using a SMART^™^ cDNA amplification kit (Clontech). Reverse transcription–polymerase chain reactions were performed using the following gene-specific primers (CYP321A7-F: 5′-ATGTTATTCCTACCATTAAGTCTTATAGCAG-3′, CYP321A7-R: 5′-TTAAATGTTTCTCGGAATGATTTGCACATCGA-3′; CYP321A9-F: 5′-ATGGTATTATTACCATTAACATTAGTACTAG-3′, CYP321A9-R: 5′-CTATTTAATTGCTCGAGGAATCAACTCAAAA-3′). The amplification conditions were as follows: 95 °C for 3 min, followed by 30 cycles (95 °C for 45 s, 55 °C for 60 s and 72 °C for 90 s) and a final extension step at 72 °C for 10 min. The PCR products were cloned into the pGEM-T Easy Vector (Promega Inc., Beijing, China) and sequenced with an ABI 377 capillary automated DNA sequencer.

### 4.4. Bioinformatic Analysis

Molecular weights and theoretical isoelectric points of the predicted CYP321A7 and CYP321A9 proteins were calculated with programs available on the ExPASy Proteomics Server (available online: http://cn.expasy.org/tools/pi_tool.html). Signal peptide sequences were predicted using the SignalP 4.1 program (available online: http://www.cbs.dtu.dk/services/SignalP/). ClustalX 1.83 was used for multiple alignments of CYP321A7 and CYP321A9 with related amino acid sequences, which were obtained by a protein BLAST search on the NCBI homepage (available online: https://blast.ncbi.nlm.nih.gov/Blast.cgi). Phylogenetic analysis was performed with the MEGA 6.0 software (neighbor-joining method; 1000 bootstrap replications).

### 4.5. Plant Allelochemicals

Jasmonic acid (≥97%), salicylic acid (≥99%), methyl jasmonate (≥98%), methyl salicylate (≥99%), aflatoxin B1 (≥98%), cinnamic acid (≥98), quercetin (≥98%), coumarin (≥99%) and xanthotoxin (≥98%) were purchased from Sigma-Aldrich (St. Louis, MO, USA). Flavone (≥98%) was obtained from Pubo Instrument Co., Ltd. (Guangzhou, China). Chemical structures of these chemicals are shown in Table S2. These chemicals represent natural allochemicals in host plants of *S. litura*. Jasmonic acid, salicylic acid, methyl jasmonate and methyl salicylate are important signal molecules that induce plant defense against insect herbivores and microbial pathogens [23,26]. Coumarin (e.g., in *Fragaria chiloensis* and *Prunus pseudocerasus*), flavone (e.g., in *Arachis hypogaea* and *Apium graveolens*) and quercetin (e.g., in *Citrus sinensis*, *Lycopersicon esculentum*) are typical allelochemicals of plants frequently eaten by *S. litura* larvae [6,34]. Aflatoxin B1, a fungal toxin produced by *Aspergillus flavus* and *A. parasiticus*, is widely distributed in many crop species [35]. Xanthotoxin (e.g., in *Foeniculum vulgare* and *Illicium verum*) and cinnamic acid (e.g., in *Cinnamomum cassia* and *Lycium barbarum*) are found in plants that are rarely attacked by *S. litura* larvae [6,34].

### 4.6. Expression of *CYP321A7* and *CYP321A9* in Different Tissues

Seven tissues of *S. litura* (cuticle, brain, midgut, fat body, Malpighian tubule, ovary and hemocytes) were dissected at day 2 from fourth instar larvae. All tissue samples were frozen in liquid nitrogen and RNA was extracted as described above. Three independent biological replicates were used for RT-qPCR analysis.

### 4.7. Stage-Dependent Expression Pattern of *CYP321A7* and *CYP321A9*

*S. litura* samples were collected at day 2 from different developmental stages (i.e., eggs and the whole bodies of first to sixth instar larvae, pupae and adults)*.* Samples were frozen in liquid nitrogen were then used for RNA extraction. RT-qPCR analysis was performed for three independent biological replicates.

### 4.8. Effects of Different Plant Allelochemicals on *CYP321A7* and *CYP321A9* Expression

Twenty synchronously developing 2-day-old fourth instar larvae of *S. litura* were offered an artificial diet supplemented with a given allelochemical at the following concentrations: 1.0 μg·g^−1^ aflatoxin B1, 0.25 mg·g^−1^ xanthotoxin, 1.0 mg·g^−1^ coumarin, 1.0 mg·g^−1^ flavone, 1.0 mg·g^−1^ quercetin, 1.0 mg·g^−1^ cinnamic acid, 2.9 μg·g^−1^ jasmonic acid, 12.0 μg·g^−1^ salicylic acid, 2.9 μg·g^−1^ methyl jasmonate and 12.0 μg·g^−1^ methyl salicylate [5,6,23,35]. The fourth instar larvae were voracious and therefore most suitable for diet experiments. Twenty control larvae were fed with an artificial diet without any supplement. After the larvae had consumed the diet for 48 h, the midgut and fat body of the larvae were dissected and frozen immediately. For expression analysis by RT-qPCR, three RNA samples per treatment (three independent biological replicates) were prepared.

### 4.9. RT-qPCR Analysis

RT-qPCR was used for expression analysis of *CYP321A7* and *CYP321A9*. Total RNA was isolated from tissue with Trizol Reagent, and examined by agarose gel electrophoresis and spectrophotometer analysis. Extracted RNAs were treated with DNase I (Thermo Fisher Scientific) and 1 μg of total RNA was reverse transcribed with the ThermoScript™ RT-PCR System kit (Thermo Fisher Scientific) following the manufacturer’s instruction. The relative expression levels of *CYP321A7* and *CYP321A9* were quantified by RT-qPCR, using gene specific primers (CYP321A7F (5′-GCAAGAGAACAAGAAAAGGTAA-3′) and CYP321A7R (5′-CAGCAGTAAAGAAGAACAAAGC-3′) for *CYP321A7*; CYP321A9F (5′-TGATGTTCTGTAATGCTGCTGT-3′) and CYP321A9R (5′-GCTCTATCTCGTATCCTGTTGT-3′) for *CYP321A9*). The β-actin primers β-actinF (5′-TGAGACCTTCAACTCCCCCG-3′) and β-actinR (5′-GCGACCAGCCAAGTCCAGAC-3′) were used for control reactions to normalize the transcript abundance among different samples [19].

RT-qPCR reactions were performed on the MJ Research Opticon instrument (Bio-Rad, Inc., Hercules, CA, USA) in a volume of 25 μL using SYBR Green I Master Mix (Roche Diagnostics Corp., Indianapolis, IN, USA) with the following thermal program: 95 °C for 15 s, followed by 40 cycles of 95 °C for 10 s, 60 °C for 30 s. After PCR, the homogeneity of the PCR product was confirmed by a melting curve analysis. The ratios of the P450 gene/β-actin gene values were calculated according to the 2^−ΔΔ*C*t^ method [36]. Three replicates were performed for each reaction.

### 4.10. Statistical Analysis

All RT-qPCR data were analyzed with the SPSS 13.0 Software Package (SPSS Inc., Chicago, IL, USA) using normalized expression levels of the *CYP321A7* and *CYP321A9* genes. Statistically significant differences (*p* < 0.05) were obtained by one-way ANOVA followed by the Duncan’s multiple range test.

## Supplementary Materials

The following are available online at www.mdpi.com/1422-0067/18/11/2278/s1.
