# Supplementary material for: Identification of Two Cytochrome Monooxygenase P450 Genes, CYP321A7 and CYP321A9, from the Tobacco Cutworm Moth (Spodoptera Litura) and Their Expression in Response to Plant Allelochemicals
Source: Int J Mol Sci. 2017 Oct 30;18(11):2278. doi: 10.3390/ijms18112278 (PMC5713248; doi:10.3390/ijms18112278)
Supplement: Supplementary file 1 [file ijms-18-02278-s001.pdf]

Table S1. Chemical structures of plant allelochemicals used in this study.

Name	Chemical structure
Jasmonic acid	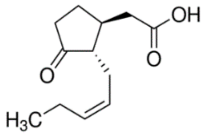
Salicylic acid	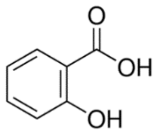
Methyl jasmonate	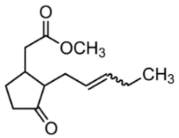
Methyl salicylate	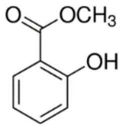
Aflatoxin B1	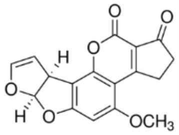

Cinnamic acid

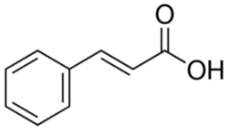

Quercetin

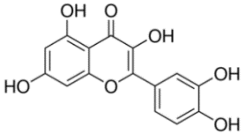

Coumarin

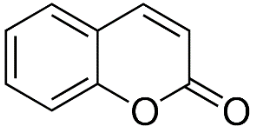

Xanthotoxin

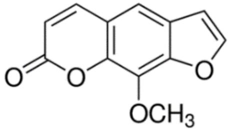

Flavone

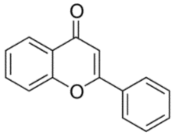

Table S2. Information on fragments of P450, esterase and glutathione S-transferase genes obtained from transcriptome analysis.

Gene fragment (bp)	Most related sequence	Predicted enzyme
406	NP_001077078.1,[a] cytochrome P450 CYP18A1,[b] *Bombyx mori*[c] (79.8% identity)[d]	P450
199	ACM66924.1, cytochrome P450 CYP314A1, *Spodoptera littoralis* (100% identity)	P450
569	AGO62005.1, cytochrome P450 CYP321A7, *Spodoptera frugiperda* (93.5% identity)	P450
485	AGO62007.1, cytochrome P450 CYP321A9, *Spodoptera frugiperda* (95.6% identity)	P450
180	AFP20609.1, cytochrome P450 CYP333A6, *Spodoptera littoralis* (81.1% identity)	P450
513	AFP20610.1, cytochrome P450 CYP333B3, *Spodoptera littoralis* (95.7% identity)	P450
492	AID55432.1, cytochrome P450 CYP337B5, *Spodoptera frugiperda* (90.9% identity)	P450
225	AID54908.1, cytochrome P450 CYP9AJ3, *Helicoverpa armigera* (85.8% identity)	P450
493	AFP20598.1, cytochrome P450 CYP4G75, *Spodoptera littoralis* (94.9% identity)	P450
165	AID54879.1, cytochrome P450 CYP4L5, *Helicoverpa armigera* (80.6% identity)	P450
306	AID54880.1, cytochrome P450 CYP4M10v2, *Helicoverpa armigera* (79.1% identity)	P450
495	AFP20600.1, cytochrome P450 CYP4S8v1, *Spodoptera littoralis* (90.5% identity)	P450
511	AGO62002.1, cytochrome P450 CYP6AB12, *Spodoptera frugiperda* (86.1% identity)	P450
504	AFP20585.1, cytochrome P450 CYP6AN4, *Spodoptera littoralis* (97.0% identity)	P450
486	AFP20591.1, cytochrome P450 CYP6AB31, *Spodoptera littoralis* (98.8% identity)	P450
503	AID55428.1, cytochrome P450 CYP6AE44 , *Spodoptera frugiperda* (80.3% identity)	P450
406	AFP20587.1, cytochrome CYP6B48, *Spodoptera littoralis* (94.3% identity)	P450
504	ADA68174.1, cytochrome P450 CYP 6B50, *Spodoptera litura* (100.0% identity)	P450
497	BAG71410.1, cytochrome P450 CYP9A9, *Spodoptera exigua* (81.1% identity)	P450
198	AID55430.1, cytochrome P450 CYP9A59, *Spodoptera frugiperda* (93.4% identity)	P450
533	ACM45975.1, cytochrome P450 CYP306A1, *Spodoptera littoralis* (96.8% identity)	P450
212	AID55431.1, cytochrome P450 9A60, *Spodoptera frugiperda* (92.9% identity)	P450

502	AFP20594.1, cytochrome P450 CYP6AE50, *Spodoptera littoralis* (97.6% identity)	P450
215	XP_004925746.1, esterase FE4-like, *Bombyx mori* (64.6% identity)	esterase
538	NP_001104822.1, alpha-esterase 45, *Bombyx mori* (70.5% identity)	esterase
455	NP_001121784.1, alpha-esterase 25, *Bombyx mori* (71.8% identity)	esterase
214	EHJ63341.1, alpha-esterase 3, *Danaus plexippus* (70.2% identity)	esterase
126	NP_001292457.1, esterase B1-like, *Plutella xylostella* (67.5% identity)	esterase
357	ABD62775.1, esterase, *Chilo suppressalis* (60.4% identity)	esterase
210	AIH07600.1 , glutathione S-transferase Theta 1, *Spodoptera litura* (90.7% identity)	glutathione S-transferase
212	AIH07599.1, glutathione S-transferase Zeta 2, *Spodoptera litura* (100.0% identity)	glutathione S-transferase
282	AIH07601.1, glutathione S-transferase Omega 2, *Spodoptera litura* (100.0% identity)	glutathione S-transferase

[a] Database accession number

[b] Gene name

[c] Species name

[d] Identity of nucleotides (expressed in %)
